# Exploring the Experiences of the NCL CAMHS Co-Production Experts by Experience in Barnet, Enfield and Haringey Mental Health Trust: A Thematic Analysis

**DOI:** 10.1192/bjo.2024.498

**Published:** 2024-08-01

**Authors:** Kiran Nijabat

**Affiliations:** Barnet Hospital, Barnet, United Kingdom

## Abstract

**Aims:**

This study focuses on the North Central London Child and Adolescent Mental Health Services (NCL CAMHS) Co-production workstream, initiated to establish co-production as a foundational method for service planning and delivery in the NCL region.

To understand what the CAMHS experts by experience members found useful and did not find useful in co-production projects within Barnet Enfield and Haringey Mental Health NHS Trust and NCL wide co-production.

**Methods:**

Semi-structured interviews conducted with experts by experience within the Barnet Enfield and Haringey (BEH) NHS Trust aimed to explore their co-production experiences, identifying facilitators and barriers. The study employed an inductive thematic analysis, grounded in a constructionist epistemological position, to analyse qualitative responses from semi-structured interviews. Braun and Clarke's (2006) methodology guided the analysis, consisting of six phases. The researchers emphasized reflexivity, reflection, and maintaining coherence, consistency, and flexibility throughout the recursive process. The voices of the lived experience co-production members played a central role in the research, influencing the entire report.

Two members of the NCL CAMHS lived experience group served as “Lived Experience Researchers” and received training on coding reliability based on Braun and Clarke's (2006) guidance.

**Results:**

Thematic analysis revealed several key findings. Recognition of co-production values within the group highlighted the importance of giving voice to service users, valuing their individual experiences, and promoting power-sharing. Facilitators included good team working, valuing diversity, accessible online sessions, and promoting equality through interactions. Conversely, barriers included inconsistent meeting timings, power imbalances, and a consultation-style dominance. Participants expressed the need for more involved projects and recommended a transformation of BEH's co-production strategy.

**Conclusion:**

Recommendations for BEH include a comprehensive evaluation of their co-production projects on the ladder of participation, emphasizing the importance of higher-level collaborations. Training for staff on co-production principles is crucial for fostering a mindset shift, and the establishment of a dedicated co-production team, including a co-production lead, is advised by service-users who co-produce. These roles can drive co-production projects, provide organizational structure, and facilitate stakeholder engagement.

